# Student’s-*t* Mixture Regression-Based Robust Soft Sensor Development for Multimode Industrial Processes

**DOI:** 10.3390/s18113968

**Published:** 2018-11-15

**Authors:** Jingbo Wang, Weiming Shao, Zhihuan Song

**Affiliations:** State Key Laboratory of Industrial Control Technology, College of Control Science and Engineering, Zhejiang University, Hangzhou 310027, China; wangjingbobo@zju.edu.cn

**Keywords:** robust soft sensor, multimode process, Student’s-*t* mixture regression, Gaussian mixture model, expectation maximization

## Abstract

Because of multiple manufacturing phases or operating conditions, a great many industrial processes work with multiple modes. In addition, it is inevitable that some measurements of industrial variables obtained through hardware sensors are incorrectly observed, recorded or imported into databases, resulting in the dataset available for statistic analysis being contaminated by outliers. Unfortunately, these outliers are difficult to recognize and remove completely. These process characteristics and dataset imperfections impose challenges on developing high-accuracy soft sensors. To resolve this problem, the Student’s-*t* mixture regression (SMR) is proposed to develop a robust soft sensor for multimode industrial processes. In the SMR, for each mixing component, the Student’s-*t* distribution is used instead of the Gaussian distribution to model secondary variables, and the functional relationship between secondary and primary variables is explicitly considered. Based on the model structure of the SMR, a computationally efficient parameter-learning algorithm is also developed for SMR. Results conducted on two cases including a numerical example and a real-life industrial process demonstrate the effectiveness and feasibility of the proposed approach.

## 1. Introduction

In industrial processes, there is a class of quality-related variables that is very important but difficult to measure, such as melt index in the polypropylene process, catalyst activation in chemical reactions, thickness of strip in the hot rolling process, octane number of gasoline, etc. Measurements of these quality variables are conventionally obtained by expensive online analyzers or time-consuming laboratory analysis, which introduces huge investment cost or large time delay [1]. Soft sensors, which are essentially mathematical models, are capable of predicting these key variables (referred to as “primary variables”) online using easy-to-measure process variables (referred to as “secondary variables”) such as flow rate, temperature, pressure, etc. Therefore, soft sensors are economical and real-time alternatives to conventional measurement of quality variables, and play an important role in process monitoring, closed-loop control, process optimization and so forth [2,3,4,5,6]. Owing to their advantages, in recent years, soft sensors have been intensively researched and extensively applied to industrial processes [7,8,9,10,11].

The methods for soft sensor modeling can generally be categorized into two groups, which are first-principle methods [12] and data-driven methods [13]. As modern industrial processes grow increasingly complex, it is difficult to obtain first-principle models. By contrast, data-driven models can be easily obtained because a large amount of process data that reflects the true operating conditions is collected in databases via field instruments [10,14]. Thus, data-driven soft sensors have gained increasing attention and popularity in real industrial processes. In the past decade, a variety of modeling algorithms have been developed and applied to construct soft sensor models. Partial least squares [15] and principle component regression [16] which are linear models for describing the relationship between quality variables and secondary variables, have been studied systematically and are widely used in real applications. Aiming at dealing with process non-linearities, soft sensors based on artificial neural networks [17] and support vector machines [18] have also been developed. Extensive reviews for the approaches and applications of soft sensors in real industrial processes can be found in [19].

Due to multiple product-grade requirements, feedstock changes, load variations, seasonal operations, etc., most industrial processes work with multiple operation modes [20]. The multimode characteristics result in process variables that are no longer Gaussian, and the functional relationship between primary and secondary variables being strongly non-linear [2], which increases the difficulty in developing high-accuracy soft sensor models. To deal with these issues, the finite mixture model (FMM) has been widely investigated and applied to real-life industrial processes. The Gaussian mixture model (GMM), which is one of the most widely adopted approaches in the FMM family, possesses the capability of approximating arbitrary unknown random distributions, including those with multiple peaks; meanwhile, GMM provides a simple and computationally efficient maximum-likelihood estimation framework by means of the expectation-maximization (EM) algorithm. Over the past few years, several studies based on GMM have been conducted for soft sensor development [13,21]. Gaussian mixture regression (GMR) treats the input space and output space together to obtain the joint probability density function (PDF) of quality and secondary variables. Then, the conditional PDF of primary variables given secondary variables can be calculated directly from their joint PDF, which can be used to derive the regression relationship between quality and secondary variables.

However, the parameter-learning procedure for the GMM is extremely sensitive to outliers, which may cause the estimated PDF of interested variables to be significantly distorted or excessive components to be required for capturing the tails of the distributions [22,23,24,25]. The outliers can be partitioned into two types, conspicuous outliers and indistinctive outliers, according to whether they are beyond the physical meaning or not. Conspicuous outliers can be easily examined and eliminated, while it is difficult to discriminate and address indistinctive outliers.

To tackle this issue, the Student’s-*t* mixture model (SMM) has been proposed as an alternative to GMM, which provides stronger robustness against outliers by means of heavier tails [26]. In the Student’s-*t* distribution, an additional parameter ν (often called degrees of freedom) compared to Gaussian distribution can be viewed as the robustness-tuning parameter. Recently, the SMM has been applied in signal/image processing applications such as human action recognition [27], medical imaging for segmentation [28], and fall detection [29], through which the SMM has achieved much better performance compared with the GMM. However, up to now, to our best knowledge, no literature has been found reporting the soft sensor based on the SMM for industrial processes. Therefore, the use of SMM for soft sensor application has not been explored. In this paper, the Student’s-*t* mixture regression (SMR) structure for the purpose of soft sensor development, which explicitly considers the functional dependency between the primary and secondary variables, is first proposed, followed by an EM algorithm-based parameter-learning algorithm for the SMR.

The rest of this paper is organized as follows. In Section 2, a brief review of the Student’s-*t* distribution and SMM are represented, followed by the elaboration of SMR as well as the procedure for parameter-learning and soft sensor development based on SMR in Section 3. In Section 4, the effectiveness and feasibility of the SMR are verified in two case studies including a numerical example and a real-life industrial process. Finally, conclusions and future work are given in Section 5.

## 2. Preliminaries

### 2.1. Student’s-t Distribution

The PDF of a *d*-dimensional Student’s-*t* distribution, with mean μ, precision matrix Λ and degree of freedom ν, is denoted as

(1) $$St\left(x|\mu ,\Lambda ,\nu \right)=\frac{{\Gamma \left(\nu /2+d/2\right)|\Lambda |}^{1/2}}{\Gamma \left(\nu /2\right){\left(\nu \pi \right)}^{d/2}}{\left(1+\frac{\Delta^{2}}{\nu }\right)}^{-(\nu +d)/2}$$

where $\Gamma \left(t\right)=\int_{0}^{\infty }z^{t-1}e^{-z}dz$ is the Gamma function, and $\Delta^{2}={\left(x-\mu \right)}^{T}\Lambda \left(x-\mu \right)$ is the squared Mahalanobis distance from x to μ.

The Student’s-*t* distribution can be viewed as an infinite mixture of scaled Gaussian distributions, i.e.,

(2) $$St\left(x|\mu ,\Lambda ,\nu \right)=\int_{0}^{\infty }N\left(x|\mu ,{\left(\eta \Lambda \right)}^{-1}\right)Gam\left(\eta |\frac{\nu }{2},\frac{\nu }{2}\right)d\eta$$

where N(·) represents the Gaussian distribution, η stands for the intermediate latent variable which is helpful for deriving the analytical solution, and Gam(·) denotes the Gamma distribution.

**Figure 1:**
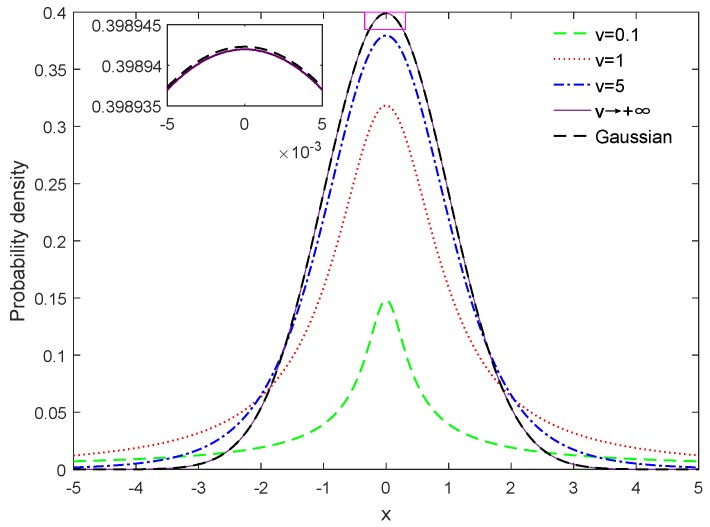
Illustration of Student’s-*t* distribution with various degrees of freedom.

Figure 1 illustrates the Student’s-*t* distribution with fixed mean vector and covariance matrix but various degrees of freedom. It can be seen that the Student’s-*t* distribution degrades the Gaussian distribution in the limit ν→+∞. Moreover, the tail of the Student’s-*t* distribution tends to be heavier when the degree of freedom ν→0. Therefore, the Student’s-*t* distribution possesses the potentiality to mitigate the adverse effect of outliers in contrast to the Gaussian distribution.

### 2.2. Student’s-t Mixture Model

Assume the secondary variable x follows the mixture distributions with *K* components as

(3) $$p\left(x|\mu ,\Lambda ,\nu ,\pi \right)=\sum_{k=1}^{K}\pi_{k}St\left(x|\mu_{k},\Lambda_{k},\nu_{k}\right)$$

where the mixing coefficients $\pi =\{\pi_{1},\pi_{2},\cdots ,\pi_{K}\}$ satisfy $\sum_{k=1}^{K}\pi_{k}=1$ together with $0⩽\pi_{k}⩽1$. In addition, let us introduce a *K*-dimensional assignment latent variable $z=(z_{1},\cdots ,z_{K})$ associated with x, in which $z_{k}$ for k=1,2,⋯,K are binary variables, i.e., $z_{k}\in \left\{0,1\right\}$. In addition, only one of the $z_{k}$ for k=1,2,⋯,K can be assigned with value 1, and the rest ones are all 0. Therefore, we have the constraint $\sum_{k=1}z_{k}=1$. If certain $z_{k}=1$, it means that the *k*-th component is responsible for generating the corresponding observed sample.

The prior distribution over z is specified in accordance with the mixing coefficients $\pi_{k}$ as

(4) $$p\left(z_{k}=1\right)=\pi_{k}$$

Using the *1-of-K* coding scheme, the prior distribution over z can also be written in the form

(5) $$p\left(z\right)=\prod_{k=1}^{K}\pi_{k}^{z_{k}}$$

Similarly, the conditional distribution of x given z is a Student’s-*t* distribution

(6) $$p\left(x|z_{k}=1\right)=St\left(x|\mu_{k},\Lambda_{k},\nu_{k}\right)$$

which can also be written as

(7) $$p\left(x|z\right)=\prod_{k=1}^{K}St{\left(x|\mu_{k},\Lambda_{k},\nu_{k}\right)}^{z_{k}}$$

## 3. Methodology

In practical applications, data collected from industrial processes are very likely to be contaminated by outliers, and it is usually non-trivial to completely remove all outliers. It has been demonstrated that the performance of GMM might be rather disappointing with the presence of outliers because the tails of the Gaussian distribution in many applications are shorter than required [22,30]. To this end, we propose the Student’s-*t* distribution mixture regression (SMR) which is detailed in this subsection.

### 3.1. Student’s-t Mixture Regression

Let us denote $X={\left\{x_{1},\cdots ,x_{N}\right\}}^{T}\in R^{N\times d}$ and $Y={\left\{y_{1},\cdots ,y_{N}\right\}}^{T}\in R^{N\times 1}$ as the input and output space of samples data, and the input variable x is assumed to be generated from Student’s-*t* distribution mixture models with *K* components as Equation (3).

**Figure 2:**
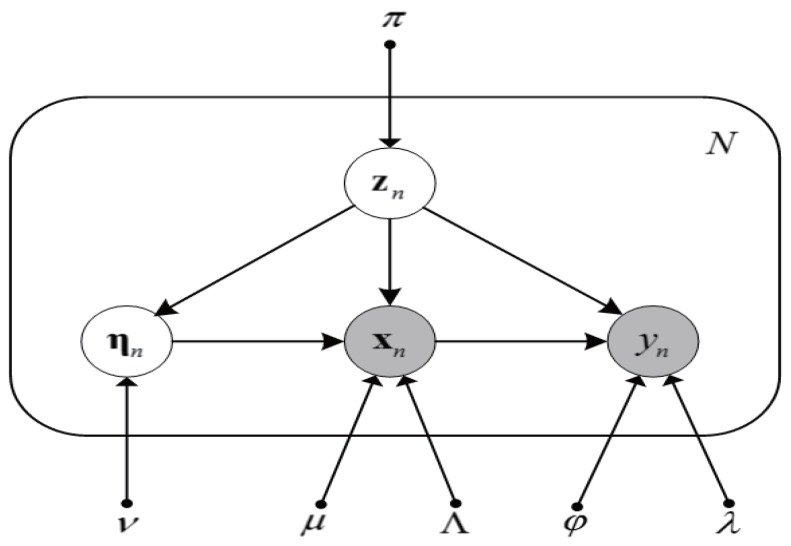
Probabilistic graphical model representation for the Student’s-*t* mixture regression model given a set of *N* independent identically distributed data points $\left\{x_{n},y_{n}\right\}$, with corresponding latent variables $\left\{z_{n},\eta_{n}\right\}$,where n=1,2,⋯,N.

The SMR is illustrated in Figure 2 in the form of a probabilistic graphical model.

For the convenience of mathematical derivation, let us define

(8) $$p\left(\eta_{nk}\right)=p\left(\eta_{n}|z_{nk}=1\right)=Gam\left(\eta_{nk}|\frac{\nu_{k}}{2},\frac{\nu_{k}}{2}\right)$$

where $\eta_{n}$ means the intermediate latent variable associated with the *n*-th sample of secondary variables (i.e., $x_{n}$). Consequently, we have

(9) $$p\left(\eta_{n}|z_{n}\right)=\prod_{k=1}^{K}Gam{\left(\eta_{nk}|\frac{\nu_{k}}{2},\frac{\nu_{k}}{2}\right)}^{z_{nk}}$$

The probability distribution over $x_{n}$ conditioned on two latent variables $z_{n}=\left(z_{n1},\cdots ,z_{nK}\right)$ and $\eta_{n}$ can be obtained as

(10) $$p\left(x_{n}|\eta_{n},z_{nk}=1\right)=N\left(x_{n}|\mu_{k},{\left(\eta_{nk}\Lambda_{k}\right)}^{-1}\right)$$

which can also be written as

(11) $$p\left(x_{n}|\eta_{n},z_{n}\right)=\prod_{k=1}^{K}N{\left(x_{n}|\mu_{k},{\left(\eta_{nk}\Lambda_{k}\right)}^{-1}\right)}^{z_{nk}}$$

For each component, linear dependence of $y_{n}$ on $x_{n}$ is introduced. Taking the single-output case for example, for k=1,⋯,K, we have

(12) $$y_{n}={\tilde{x}}_{n}^{T}\varphi_{k}+\varepsilon_{k}$$

where $\varphi_{k}$ represents the regression coefficient vector, $\varepsilon_{k}$ means zero-mean Gaussian-distributed noise variable with covariance $\lambda_{k}^{-1}$, and ${\tilde{x}}_{n}={\left[x_{n}^{T},1\right]}^{T}$.

According to Equation (12), for the *k*-th component, the conditional PDF of $y_{n}$ given $x_{n}$ can be obtained as

(13) $$p\left(y_{n}|x_{n},z_{nk}=1\right)=N\left(y_{n}|{\tilde{x}}_{n}^{T}\varphi_{k},\lambda_{k}^{-1}\right)$$

According to Equation (13), we have

(14) $$p\left(y_{n}|x_{n},z_{n}\right)=\prod_{k=1}^{K}N{\left(y_{n}|{\tilde{x}}_{n}^{T}\varphi_{k},\lambda_{k}^{-1}\right)}^{z_{nk}}$$

### 3.2. Parameters Learning for the SMR

The parameters for the SMR that need to be learnt are denoted as $\Theta ={\left\{\pi_{k},\mu_{k},\Lambda_{k},\nu_{k},\varphi_{k},\lambda_{k}\right\}}_{k=1}^{K}$. The EM algorithm, consisting of the expectation step (*E*-step) and maximization step (*M*-step), is an ideal approach to addressing the issues of missing values [31] (corresponding to the latent variables appeared in the SMR). Therefore, we adopt the EM to perform the parameter-learning task for the SMR.

In the *E* step, the posterior distribution over latent variables $z_{1},\cdots ,z_{N}$, which are collectively denoted as $Z={\left(z_{n}\right)}_{n=1}^{N}$, associated with the training dataset $\left(X,Y\right)={\left(x_{n},y_{n}\right)}_{n=1}^{N}$ can be calculated as

(15) $$\begin{array}{cc} p(z_{nk}=1|x_{n},y_{n}) & =\frac{p\left(z_{nk}=1\right)p\left(y_{n}|x_{n},z_{nk}=1\right)p\left(x_{n}|z_{nk}=1\right)}{\sum_{k=1}^{K}p\left(z_{nk}=1\right)p\left(y_{n}|x_{n},z_{nk}=1\right)p\left(x_{n}|z_{nk}=1\right)}\\ & =\frac{\pi_{k}N\left(y_{n}|{\tilde{x}}_{n}^{T}\varphi_{k},\lambda_{k}^{-1}\right)St\left(x_{n}|\mu_{k},\Lambda_{k},\nu_{k}\right)}{\sum_{k=1}^{K}\pi_{k}N\left(y_{n}|{\tilde{x}}_{n}^{T}\varphi_{k},\lambda_{k}^{-1}\right)St\left(x_{n}|\mu_{k},\Lambda_{k},\nu_{k}\right)} \end{array}$$

Therefore, the expectation of $z_{nk}$ based on the posterior distribution can be calculated as

(16) $$\begin{array}{cc} \langle z_{nk}\rangle & =p(z_{nk}=1|x_{n},y_{n})\\ & =\frac{\pi_{k}N\left(y_{n}|{\tilde{x}}_{n}^{T}\varphi_{k},\lambda_{k}^{-1}\right)St\left(x_{n}|\mu_{k},\Lambda_{k},\nu_{k}\right)}{\sum_{k=1}^{K}\pi_{k}N\left(y_{n}|{\tilde{x}}_{n}^{T}\varphi_{k},\lambda_{k}^{-1}\right)St\left(x_{n}|\mu_{k},\Lambda_{k},\nu_{k}\right)} \end{array}$$

Given the latent variable $z_{n}$ and observed variable $x_{n}$, the posterior distribution over $\eta_{n}$ can be calculated as

(17) $$\begin{array}{c} p\left(\eta_{n}|x_{n},z_{nk}=1\right)\propto p\left(x_{n}|\eta_{n},z_{nk}=1\right)p\left(\eta_{n}|z_{nk}=1\right)\\ \propto N\left(x_{n}|\mu_{k},{\left(\eta_{n}\Lambda_{k}\right)}^{-1}\right)Gam\left(\eta_{n}|\frac{v_{k}}{2},\frac{v_{k}}{2}\right)\\ \propto \eta_{n}^{\frac{d+v_{k}}{2}-1}\exp\left\{-\frac{{\left(x_{n}-\mu_{k}\right)}^{T}\Lambda_{k}\left(x_{n}-\mu_{k}\right)+v_{k}}{2}\eta_{n}\right\} \end{array}$$

Comparing the definition of the Gamma distribution, we have

(18) $$\begin{array}{cc} p(\eta_{nk}|x_{n}) & =p(\eta_{n}|x_{n},z_{nk}=1)\\ & =Gam\left(\eta_{nk}|\frac{\nu_{k}+d}{2},\frac{\nu_{k}}{2}+\frac{1}{2}{\left(x_{n}-\mu_{k}\right)}^{T}\Lambda_{k}\left(x_{n}-\mu_{k}\right)\right) \end{array}$$

Thus, we can obtain the expectations

(19) $$\langle \eta_{nk}\rangle =\frac{\nu_{k}+d}{\nu_{k}+{\left(x_{n}-\mu_{k}\right)}^{T}\Lambda_{k}\left(x_{n}-\mu_{k}\right)}$$

(20) $$\langle \ln\left(\eta_{nk}\right)\rangle =\psi \left(\frac{\nu_{k}+d}{2}\right)-\ln\left(\frac{\nu_{k}}{2}+\frac{1}{2}{\left(x_{n}-\mu_{k}\right)}^{T}\Lambda_{k}\left(x_{n}-\mu_{k}\right)\right)$$

where ψ(·) is the digamma function defined as $\psi \left(x\right)=d\left(\Gamma (x)\right)/dx$.

Subsequently, in the *M* step, with the assumption that the samples are independent and identically distributed, the expectation of complete data log-likelihood function is first formulated as

(21) $$\begin{array}{cc} L(\Theta ) & =\langle \ln p(X,Y,Z,\eta )\rangle \\ & =\langle \ln p(Y|X,Z)\rangle +\langle \ln p(X|Z,\eta )\rangle +\langle \ln p(\eta |Z)\rangle +\langle \ln p(Z)\rangle \end{array}$$

where

(22) $$\begin{array}{c} \langle \ln p\left(Y|X,Z\right)\rangle =\sum_{n=1}^{N}\sum_{k=1}^{K}\langle z_{nk}\rangle \{-\frac{1}{2}\ln\left(2\pi \right)+\frac{1}{2}\ln\left(\lambda_{k}\right)-\frac{1}{2}\lambda_{k}{\left(y_{n}-{\tilde{x}}_{n}^{T}\varphi_{k}\right)}^{2}\} \end{array}$$

(23) $$\begin{array}{c} \langle \ln p\left(X|Z,\eta \right)\rangle =\sum_{n=1}^{N}\sum_{k=1}^{K}\langle z_{nk}\rangle \{-\frac{d}{2}\ln\left(2\pi \right)+\frac{1}{2}\ln(|\Lambda_{k}\left|\right)+\frac{d}{2}\langle \ln\left(\eta_{nk}\right)\rangle -\frac{\langle \eta_{nk}\rangle }{2}{\left(x_{n}-\mu_{k}\right)}^{T}\Lambda_{k}\left(x_{n}-\mu_{k}\right)\} \end{array}$$

(24) $$\begin{array}{c} \langle \ln p\left(\eta |Z\right)\rangle =\sum_{n=1}^{N}\sum_{k=1}^{K}\langle z_{nk}\rangle \{-\ln\left(\Gamma (\frac{\nu_{k}}{2})\right)+\frac{\nu_{k}}{2}\ln\left(\frac{\nu_{k}}{2}\right)+\left(\frac{\nu_{k}}{2}-1\right)\langle \ln\left(\eta_{nk}\right)\rangle -\frac{\nu_{k}}{2}\langle \eta_{nk}\rangle \} \end{array}$$

(25) $$\begin{array}{c} \langle \ln p\left(Z\right)\rangle =\sum_{n=1}^{N}\sum_{k=1}^{K}\langle z_{nk}\rangle \ln\left(\pi_{k}\right) \end{array}$$

and $\eta ={\left(\eta_{n}\right)}_{n=1}^{N}$.

Setting the derivatives of Equation (21) with respect to $\mu_{k}$ to zero leads to

(26) $$\begin{array}{cc} \frac{\partial L(\Theta )}{\partial \mu_{k}} & =\sum_{n=1}^{N}\langle z_{nk}\rangle \langle \eta_{nk}\rangle \Lambda_{k}\left(x_{n}-\mu_{k}\right)=0\\ & \Rightarrow \mu_{k}=\sum_{n=1}^{N}\langle z_{nk}\rangle \langle \eta_{nk}\rangle x_{n}/\sum_{n=1}^{N}\langle z_{nk}\rangle \langle \eta_{nk}\rangle \end{array}$$

Similarly, we have    

(27) $$\begin{array}{c} \frac{\partial L(\Theta )}{\partial \Lambda_{k}}=\sum_{n=1}^{N}\langle z_{nk}\rangle \left\{\Lambda_{k}^{-1}-\langle \eta_{nk}\rangle \left(x_{n}-\mu_{k}\right){\left(x_{n}-\mu_{k}\right)}^{T}\right\}=0\\ \Rightarrow \Lambda_{k}^{-1}=\sum_{n=1}^{N}\langle z_{nk}\rangle \langle \eta_{nk}\rangle \left(x_{n}-\mu_{k}\right){\left(x_{n}-\mu_{k}\right)}^{T}/\sum_{n=1}^{N}\langle z_{nk}\rangle \end{array}$$

(28) $$\begin{array}{cc} \frac{\partial L(\Theta )}{\partial \lambda_{k}} & =\sum_{n=1}^{N}\langle z_{nk}\rangle \left(\lambda_{k}^{-1}-{\left(y_{n}-{\tilde{x}}_{n}^{T}\varphi_{k}\right)}^{2}\right)=0\\ & \Rightarrow \lambda_{k}=\{\sum_{n=1}^{N}\langle z_{nk}\rangle {\left(y_{n}-{\tilde{x}}_{n}^{T}\varphi_{k}\right)}^{2}/\sum_{n=1}^{N}\langle z_{nk}\rangle {\}}^{-1} \end{array}$$

Setting the derivatives of Equation (21) with respect to $\varphi_{k}$ to zero leads to

(29) $$\begin{array}{cc} \frac{\partial L(\Theta )}{\partial \varphi_{k}} & ={\tilde{X}}^{T}R_{k}\tilde{X}\varphi_{k}-{\tilde{X}}^{T}R_{k}Y=0\\ & \Rightarrow \varphi_{k}={\left({\tilde{X}}^{T}R_{k}\tilde{X}\right)}^{-1}{\tilde{X}}^{T}R_{k}Y \end{array}$$

where $R_{k}=diag\left(\langle z_{1k}\rangle ,\langle z_{1k}\rangle ,\cdots ,\langle z_{nk}\rangle \right)$, $\tilde{X}=\left[X,1\right]$, 1 is the column with all element 1.

The parameter $\nu_{k}$ can be obtained by solving the non-linear equation as follows.

(30)∂L(Θ)∂νk=∑n=1N〈znk〉{−ψ(νk2)+ln(νk2)+1+〈ln(ηnk)〉−〈ηnk〉}=0(31)⇒−ψ(νk2)+ln(νk2)+1+∑n=1N〈znk〉〈ln(ηnk)〉−〈ηnk〉∑n=1N〈znk〉=0

Please note that it has been proved that the left-hand side of Equation (31) strictly decreases from +∞ to a minus value as $\nu_{k}$ increases in (0,+∞) [32]. Therefore, solving Equation (31) for $\nu_{k}$ is not difficult by the means of many one-dimensional search methods, such as the dichotomy method.

Using the constraint $\sum_{k=1}^{K}\pi_{k}=1$ and introducing the Lagrange multiplier γ, we can obtain

(32) $$\left\{\begin{array}{c} \frac{\partial \tilde{L}\left(\Theta \right)}{\partial \pi_{k}}=\sum_{n=1}^{N}\langle z_{nk}\rangle /\pi_{k}+\gamma =0\\ \sum_{k=1}^{K}\pi_{k}=1 \end{array}\right.\Rightarrow \pi_{k}=\sum_{n=1}^{N}\langle z_{nk}\rangle /N$$

where $\tilde{L}\left(\Theta \right)=L\left(\Theta \right)+\gamma \left(\sum_{k=1}^{K}\pi_{k}-1\right)$.

In the light of the updated equations such as the derivation above, the robustness of SMR compared with GMR can be clearly seen with the use of degrees of freedom ν. As the degrees of freedom parameter ν is introduced, the outliers with large Mahalanobis distance have small value of the expectation of $\eta_{nk}$ as can be drawn from Equation (19), resulting in the outliers being down-weighted and the influence of outliers on parameters estimation being significantly reduced. Taking the precision matrix of each component, for example, based on GMR the updated equation will be converted into $\Lambda_{k}^{-1}=\sum_{n=1}^{N}\langle z_{nk}\rangle \left(x_{n}-\mu_{k}\right){\left(x_{n}-\mu_{k}\right)}^{T}/\sum_{n=1}^{N}\langle z_{nk}\rangle$, which means that the data’s outliers will highly influence the estimates. However, taking this example to the extreme, the outliers which are extremely different to the majority of dataset are down-weighted to zero because in the SMR the $\langle \eta_{nk}\rangle$ associated with the outliers will be zero, resulting in the influence of outliers on precision matrix estimates being removed.

As the model above-mentioned parameters are updated by iterative learning, the iterative process terminates when L(Θ) converges, and the convergence criterion can be defined as

(33) $$|\frac{L\left(\Theta_{t}\right)-L\left(\Theta_{t-1}\right)}{L(\Theta_{t-1})}|<\varepsilon$$

where $L(\Theta_{t})$ denotes the value of L(Θ) at the *t*th iteration and ε represents the threshold value, which is specified by the user.

Up to now, we can summarize the detailed procedure for training the SMR in Algorithm 1.

**Algorithm 1** Pseudocode for training SMR.     Given *K*, initialize $\Theta ={\left\{\pi_{k},\mu_{k},\Lambda_{k},\nu_{k},\varphi_{k},\lambda_{k}\right\}}_{k=1}^{K}$, and the maximumiterationtimes;     Set t=0;     **while**
t<maximumiterationtimes
**do**          Set t=t+1;          **for** k=1,⋯,K; n=1,⋯,N
**do**             Calculate $\langle z_{nk}\rangle$ using Equation (16);             Calculate $\langle \eta_{nk}\rangle$ and $\langle \ln\eta_{nk}\rangle$ using Equation (19) and Equation (20), respectively;          **end for**          **for**
k=1,⋯,K
**do**             Update $\mu_{k}$, $\Lambda_{k}$, $\lambda_{k}$ and $\varphi_{k}$, $\pi_{k}$ with Equation (26), Equation (27), Equation (28), Equation (29)             and Equation (32) respectively;             Solve Equation (31) for $\nu_{k}$;          **end for**          Calculate L(Θ) using Equation (21).          **if** the convergence criterion in Equation (33) is satisfied **then**                Terminate **while**;          **end if**       **end while**

### 3.3. Soft Sensor Development Based on SMR

Based on the SMR, a soft sensor model can be easily developed for predicting the quality variable $y_{q}$ when a sample $x_{q}$ of process variables is available.

To begin with, the posterior distribution of the associated latent variable $z_{q}=\left(z_{q1},\cdots ,z_{qK}\right)$ is calculated as

(34) $$\begin{array}{cc} p(z_{qk}=1|x_{q}) & =\frac{p\left(x_{q}|z_{qk}=1\right)p\left(z_{qk}=1\right)}{\sum_{k=1}^{K}p\left(x_{q}|z_{qk}=1\right)p\left(z_{qk}=1\right)}\\ & =\frac{\pi_{k}St\left(x_{q}|\mu_{k},\Lambda_{k},\nu_{k}\right)}{\sum_{k=1}^{K}\pi_{k}St\left(x_{q}|\mu_{k},\Lambda_{k},\nu_{k}\right)}≜R_{qk} \end{array}$$

Subsequently, the probability distribution $y_{q}$ conditioned on $x_{q}$ can be obtained as

(35) $$\begin{array}{cc} p(y_{q}|x_{q}) & =\sum_{k=1}^{K}p\left(y_{q}|x_{q},z_{qk}=1\right)p\left(z_{qk}=1|x_{q}\right)\\ & =\sum_{k=1}^{K}R_{qk}N\left(y_{q}|{\tilde{x}}_{q}^{T}\varphi_{k},\lambda_{k}^{-1}\right) \end{array}$$

Finally, the prediction of $y_{q}$ can be obtained as

(36) $${\hat{y}}_{q}=\sum_{k=1}^{K}R_{qk}{\tilde{x}}_{q}^{T}\varphi_{k}$$

## 4. Case Studies

In this section, the proposed method is first evaluated using a numerical example and then applied to develop soft sensors for an industrial primary reformer in an ammonia synthesis plant [33]. For comparison purposes, the performance of multiple dynamic PLS (Multi-DPLS) [34,35] and GMR are also provided as benchmarks. Please note that the Multi-DPLS is realized by first referring to the work in [34], where the GMM is used for data clustering, followed by constructing a sub-PLS model for each data cluster. Then, we extend the PLS model to the DPLS model by augmenting the input vector according to [35].

The root mean squares error (RMSE) is used to evaluate the prediction accuracies of various methods, which is defined as

(37) $$\mathrm{RMSE}=\sqrt{\sum_{n=1}^{N_{t}}{\left(y_{n}-{\hat{y}}_{n}\right)}^{2}/N_{t}}$$

where $y_{n}$ and ${\hat{y}}_{n}$ are the true value and predicted value of quality variable, respectively, and $N_{t}$ is the size of the testing dataset.

To deal with the influence of randomness of initial parameters, a total of 100 simulations are carried out for both the GMR and SMR, and their final parameters are selected as those that can minimize the RMSE on the validating dataset, while the generalization performance of various methods are evaluated on the testing dataset. The configurations of the used computer are given as follows: CPU: Core i5-4570 (3.2 GHz × 2), RAM: 8 GB, OS: Windows 10, and Software: MATLAB (R2016b). The CPU time (CPT) spent in offline model training (CPT${}_{trn}$, in seconds) and in online predicting (CPT${}_{tst}$, in seconds) are employed to assess the computational efficiency for different methods. In both case studies, the threshold values for diagnosing the convergence for the SMR and GMR are set as $10^{-6}$.

### 4.1. Numerical Example

**Figure 3:**
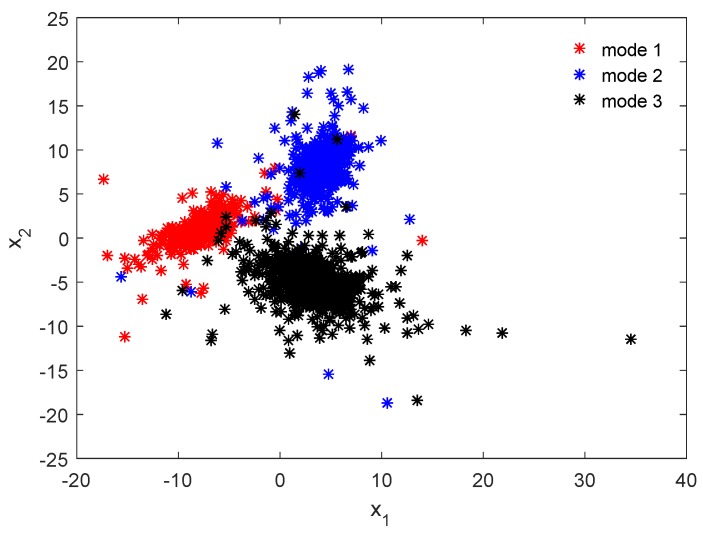
Visualization of the data distribution in the input space.

We assume a 2-dimensional input variables $x={\left(x_{1},x_{2}\right)}^{T}$ and a scalar output *y* are generated from a mixture of three Student’s-*t* distributions based on Equations (3) and (12), in which the configurations of each component are listed in Table 1. Please note that as the non-diagonal elements for the precision matrices $\Lambda_{k}$ are not zero, the correlations among the input variables are taken into consideration, which can be captured by the proposed model using Equation (3). In addition, in our model setting, the vector $x={\left(x_{1},x_{2}\right)}^{T}$ is assumed to obey a mixture of multivariate Student’s-*t* distributions, and we do not need to build one SMM for each of variable. Figure 3 illustrates the data distributions from the input space, which clearly shows the multimode characteristics.

In the simulation, three datasets, namely the training dataset, validating dataset, and testing dataset, each of which consists of 2000 samples, were generated. The training dataset is used for parameter learning, while the validating dataset is used for determining the initialized values of model parameters for the Multi-DPLS, GMR, and SMR models. In this example, the number of mixing components for Multi-DPLS, GMR, and SMR models were set as 3 in advance; in addition, the dimensionality of the latent space for each sub-PLS model in the Multi-DPLS was set as 2. The performance of various methods are evaluated on the testing dataset, which is unseen at the training stage. Moreover, 1%, 3% and 5% outliers are randomly added into the input data samples, respectively.

According to the proportion of the sample number of each mode, the outliers are generated by transforming a certain coordinate of some sample data randomly selected to the value far away from its center. For example, 3% rate outliers are added to the training dataset containing 2000 samples, which is to say there are 12, 18, and 30 outliers added to each mode, respectively [36].

By using trial and error, the order of the Multi-DPLS is determined as 4, i.e., the values of input variables in the past four moments are also used to estimate the value of the current output. Recall that in this case, data samples were generated independently with each other without dynamics. The reason the Multi-DPLS with the order of 4 achieves the best performance can be explained as follows. The augmented input vector is helpful at improving the classification accuracy for the GMM, because the samples at some augmented sampling instances may be located at non-overlapped areas among the three modes; meanwhile, the PLS can deal with the data-collinearity. That is why the performance of the Multi-DPLS gets enhanced when the order increases. However, as the order further increases, the dimensionality of the input vector significantly increases, too, which leads to inaccurate estimations of the probability density functions. That is why performance of the Multi-DPLS deteriorates when the order is greater than 4.

**Figure 4:**
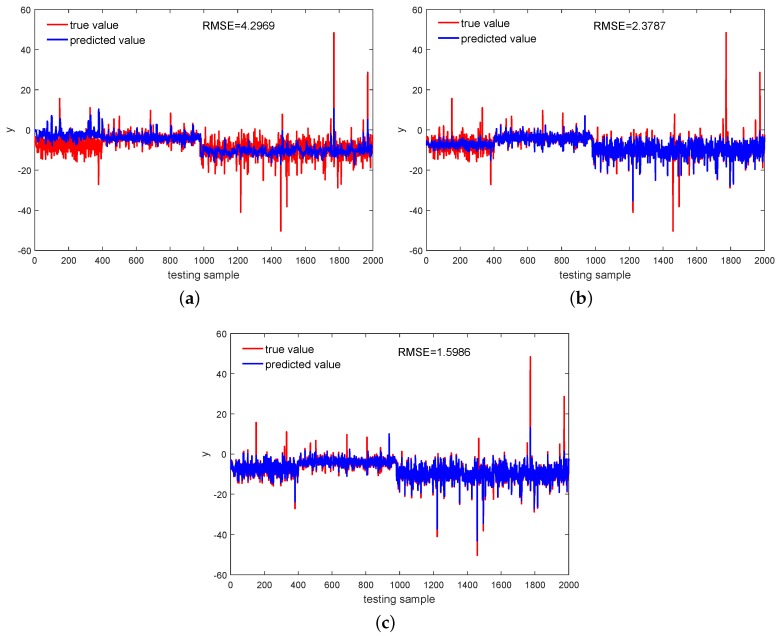
With 3% rate outliers, predictions for the output variable achieved by: (**a**) Multi-DPLS, (**b**) GMR, (**c**) SMR.

Predictions for *y* by the models based on the Multi-DPLS, GMR, and SMR with the outlier rate set as 3% are visualized in Figure 4, from which for the Multi-DPLS large deviations existing in the first mode and third mode can be clearly found. This is because the information of output space in the mode identification step is ignored, and then the performance of clustering the high-dimensional data is rather unsatisfactory, leading to a PLS model built into each mode that cannot explain the true functional dependency between the output and input variables well. In contrast, the GMR and SMR-based models, which treat the input space and output space together, are more powerful at modeling the multimode process. However, intuitively, we can recognize that the SMR performs better compared with the GMR in terms of predicting samples from the first mode.

For more in-depth analyses, predictive accuracies of three methods on the validating dataset and testing dataset are quantified in Table 2. As can be seen, the performance of the Multi-DPLS model is rather disappointing, while the predictive accuracies of the GMR and SMR models are much higher. In addition, one can see that as the number of outliers increases, the performances of both the GMR and SMR-based models deteriorate. However, the deteriorations for the SMR-based model are much slighter compared with those for the GMR-based model. To be specific, as the outlier rate rises from 1% to 3% and 5%, the generalization RMSE for the GMR-based model is increased by 41.8% and 63.8%, respectively; in contrast, the increment of generalization RMSE for the SMR-based model is only 5.1% and 7.7%, respectively, which demonstrate that the SMR-based model is much more robust against outliers compared with the GMR-based model.

**Figure 5:**
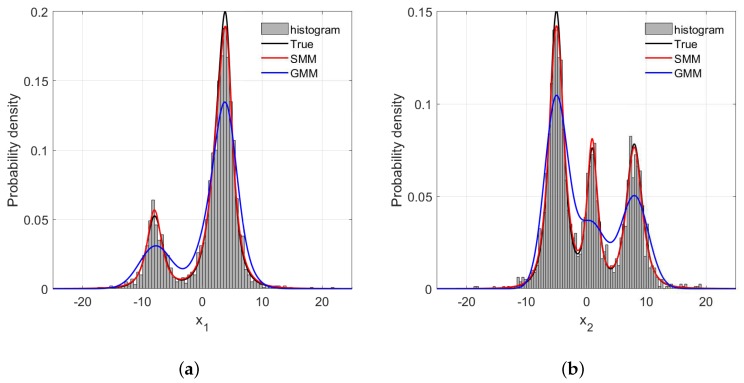
The frequency histogram and probability density curve with 1% rate outliers: (**a**) $x_{1}$ direction; (**b**) $x_{2}$ direction.

**Figure 6:**
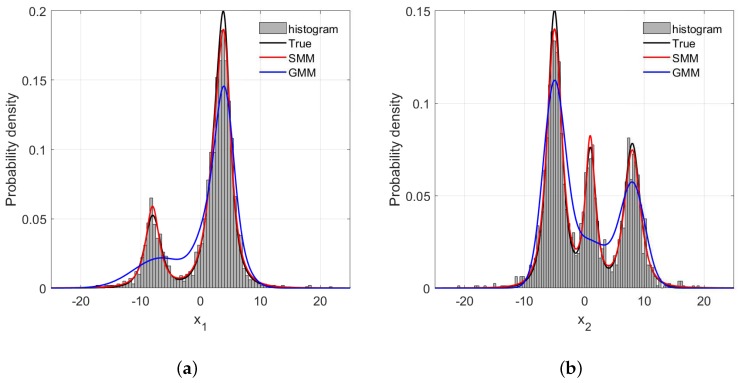
The frequency histogram and probability density curve with 3% rate outliers: (**a**) $x_{1}$ direction; (**b**) $x_{2}$ direction.

**Figure 7:**
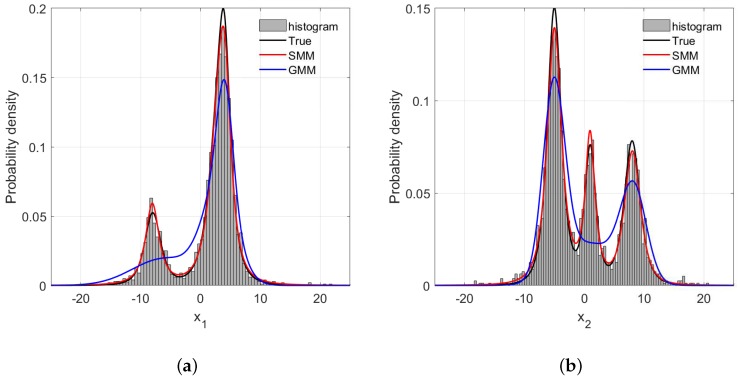
The frequency histogram and probability density curve with 5% rate outliers: (**a**) $x_{1}$ direction; (**b**) $x_{2}$ direction.

For probabilistic methods such as the GMR and SMR, correctly estimating the PDFs of process variables is a prerequisite to high predictive accuracy. In this synthetic case, the estimations of PDFs of $x_{1}$ and $x_{2}$ with different amounts of outliers are illustrated in Figure 5, Figure 6 and Figure 7. One can readily recognize that due to the long tails of data distributions, the PDFs of $x_{1}$ and $x_{2}$ estimated by GMR have been significantly skewed compared with the data histograms and true PDFs. In addition, such distortion becomes more severe as the number of outliers increase. In particular, the GMR basically fails to capture the middle peak from the $x_{2}$ direction. By contrast, the PDFs estimated by the SMR fit the data histograms well, and are barely affected by the increase of outliers, which is the reason that the SMR-based model can provide satisfactory performance with various numbers of outliers.

For the numerical example the time consumed by these three methods are listed in Table 3.

It is easily seen from Table 3 that the differences between CPT${}_{tst}$ for these three methods can be negligible. The CPT${}_{trn}$ for the Multi-DPLS and GMR are comparable. Please note that in the SMR the parameter $\nu_{k}$ is estimated by solving a non-linear equation with the help of the dichotomy method, which results in more time for iterative learning. However, the computational efficiency for a soft sensor based on SMR is still acceptable.

### 4.2. Primary Reformer

**Figure 8:**
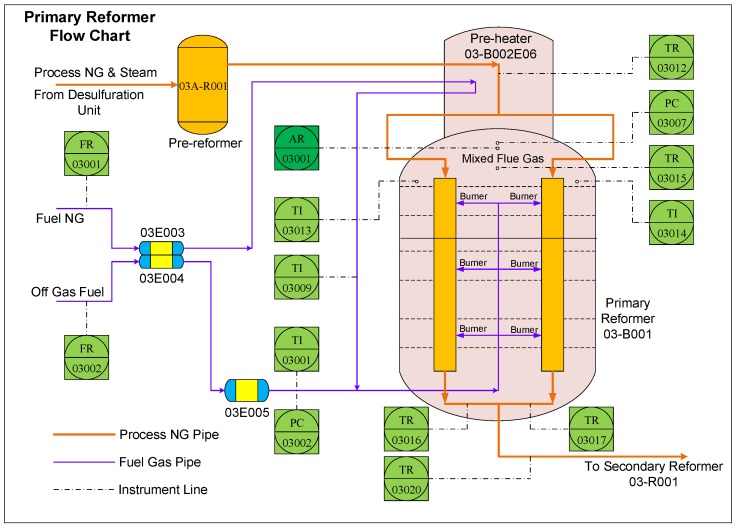
Flowchart of the primary reformer.

The primary reformer is an important part of hydrogen-manufacturing units in the ammonia synthesis process for producing NH_3_, which is the main material in the urea synthesis process. The flowchart of the primary reformer is illustrated in Figure 8.

Main transformation reactions set off in the primary reformer are

(38) $$\begin{array}{c} C_{n}H_{2n+2}+nH_{2}O\;\overset{\Delta }{↔}\;nCO+\left(2n+1\right)H_{2}\\ CH_{4}+H_{2}O\;\overset{\Delta }{↔}\;CO+3H_{2}\\ CO+H_{2}O\;\overset{\Delta }{↔}\;CO_{2}+H_{2} \end{array}$$

According to the reaction mechanism, the temperature in the furnace plays a significant role in the purity of hydrogen; thus, the temperature should be strictly monitored and controlled, which is realized by manipulating the burning conditions at the dense burner. One of the effective approaches to stabilizing the burning condition is to control the oxygen concentration in the furnace at the specified interval. However, the measurement of oxygen concentration (i.e., the quality-related variable for the primary reformer) in practice is expensive, due to an exorbitant mass spectrometer, or time-consuming, due to offline laboratory analysis, both of which fail to satisfy the requirement of real-time control and production. To cope with this issue, a soft sensor based on a historical dataset is desirable for online estimation of the oxygen concentration, which is illustrated with a dark green block in Figure 8.

Based on expert knowledge of process mechanisms and experiences from engineers, 13 process variables, including pressures and temperatures, are selected as secondary variables for soft sensor modeling, which are illustrated with light-green blocks in Figure 8. Detailed descriptions of these secondary variables are presented in Table 4.

**Figure 9:**
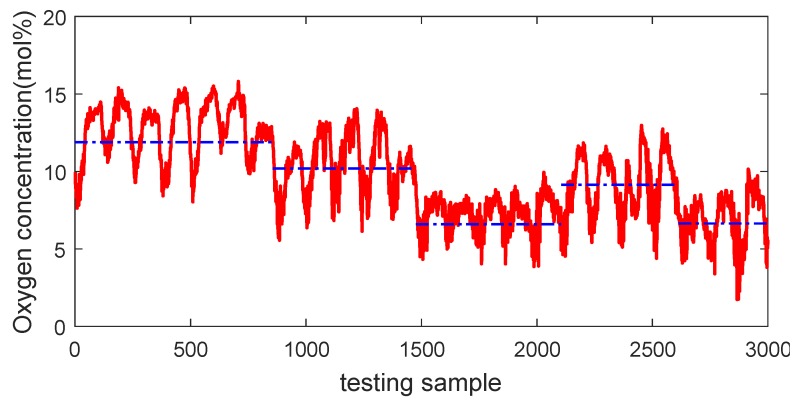
Visualization of multimode characteristics of the primary reformer.

A total of 7000 samples recorded from January 2015 to July 2015 were collected from the database of distributed control systems of a real-world primary. The collected samples are evenly partitioned into three parts, i.e., 2000 samples serve as the training dataset, 2000 samples are used as the validating dataset for model selection, and the remaining 3000 samples constitute the testing dataset for evaluating the generalization performance of various soft sensors. By taking the testing samples, for example, it is obvious that the process basically involves five large operating conditions, as shown by the dash-dot blue line in Figure 9, which indicates that the primary reformer is characterized by multiple modes.

**Figure 10:**
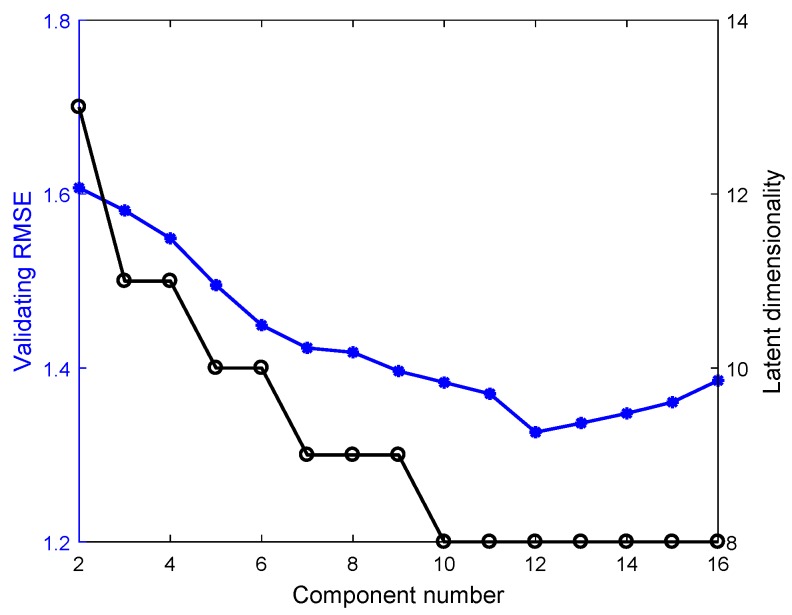
The validating RMSE and latent dimensionality based on Multi-DPLS.

**Figure 11:**
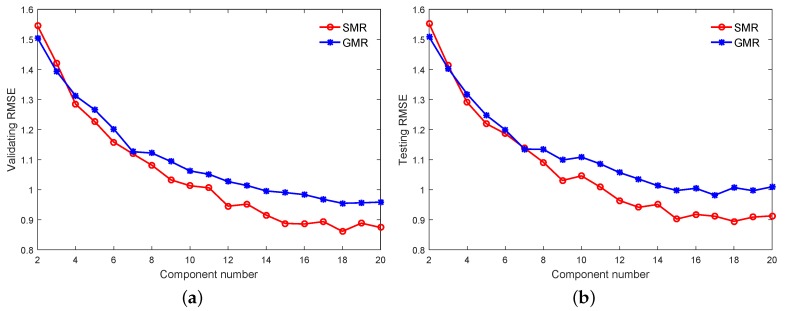
The RMSE on: (**a**) the validating datasets, (**b**) the testing datasets.

As with the numerical example, the order of the Multi-DPLS is determined as 3, and Figure 10 shows that the number of components and the dimensionality of latent space are determined as 12 and 8, respectively, in which the Multi-DPLS has the minimum RMSE on the validating dataset. In addition, the initial values of model parameters for the GMR and SMR-based soft sensors, as well as the model selections for them are also completed on the validating dataset. In particular, the best performances for the GMR and SMR-based soft sensors with various numbers of mixing components (i.e., *K*) are visualized in Figure 11a, which indicates that both the predictive RMSEs of GMR and SMR-based soft sensors reach the minimum at K=18. However, for the SMR-based soft sensor, we see that as K≥15, the validating RMSE almost stabilize at 0.88. Considering the fact that the larger the *K*, the higher the model complexity, we determine the optimal *K* for the SMR-based soft sensor as 15. Based on the same consideration, the optimal *K* for the GMR-based soft sensor is selected as 18. Meanwhile, for the GMR and SMR-based soft sensors, the generalization performances on the testing dataset are compared in Figure 11b.

From Figure 11a,b we can recognize that: (1) the selected optimal values of *K* and initialized model parameters upon the validating dataset can basically embody the true generalization performance upon the testing dataset for the GMR and SMR-based soft sensors; (2) upon both the validating and testing dataset, although with small values of K(≤7), the performances of the two soft sensors are comparable, and the SMR-based soft sensor starts to show apparent predictive advantage over the GMR-based one as K≥8; (3) the number of components is much larger than the number of operating conditions, because each operating condition may consist of several modes. The underlying reason for this phenomenon is that for complex processes, one Student’s-*t* distribution still does not model one operating condition well, and more Student’s-*t* distributions are required for one operating condition; and (4) the number of components is mainly determined through the division of the spatial pattern of input and output variables rather than the number of input variables, so there is no relationship between the number of components and the number of input variables in the mixture models.

**Figure 12:**
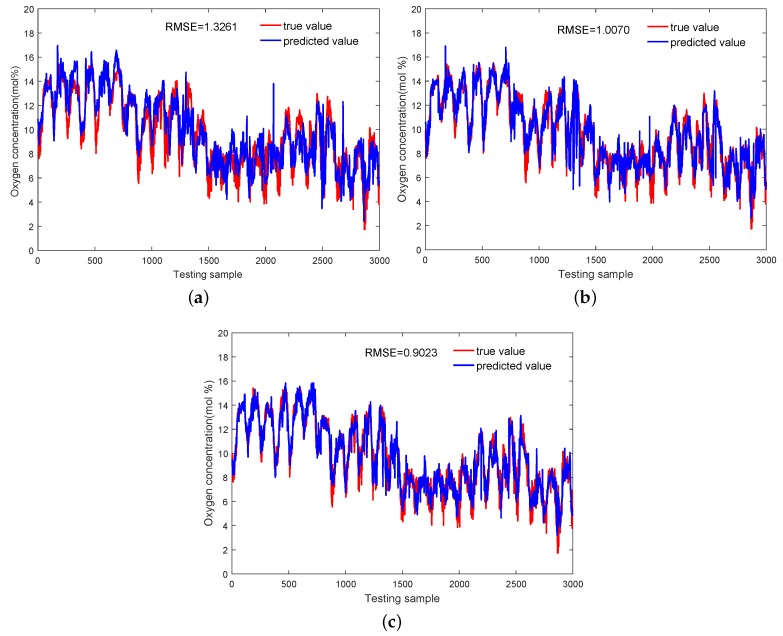
Predictions of the oxygen concentration achieved by: (**a**) Multi-DPLS, (**b**) GMR, (**c**) SMR.

**Figure 13:**
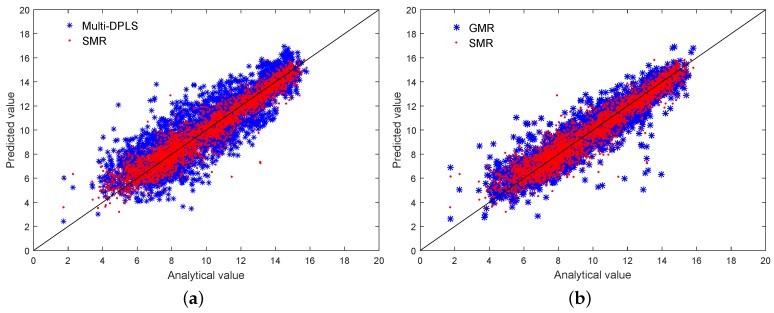
Scatter plot comparisons for estimating the concentration of O_2_: (**a**) Multi-DPLS and SMR; (**b**) GMR and SMR.

Predictions of the O_2_ concentration by soft sensors based on the Multi-DPLS, GMR, and SMR are visualized in Figure 12, where their generalization abilities are also presented in terms of RMSE. As can be seen, the Multi-DPLS model has worst performance. Except for the ignorance of output information in the mode identification, the other reason is that the augmented input vector has high dimensionality (52 dimensions in the primary reformer), resulting in an exponentially increasing number of samples being required to acquire the correct estimations of probability distribution of each mode. In contrast, both the GMR and SMR-based soft sensors, which employ mixture component models, can significantly improve the prediction performance. Scatter plot comparisons among the Multi-DPLS, GMR and SMR presented in Figure 13 could provide more insights. It can be clearly seen that the predictions obtained by the Multi-DPLS are more scattered. However, predictions of soft sensors based on GMR and SMR lean much closer to the black diagonal line, indicating higher predictive accuracy. Moreover, since the SMR takes the robustness against outliers into consideration, the predictions obtained by SMR have tighter scatters around the black diagonal line, which demonstrates the advantages of SMR compared with GMR. The predictive RMSE on the testing dataset also demonstrate that the SMR-based soft sensor has stronger generalization ability than the GMR-based one. For further quantitative analyses, the determination coefficients for the Multi-DPLS, GMR, and SMR are also calculated as 0.7729, 0.8655, and 0.9233, respectively, from which the same conclusion can be drawn.

The consumed time by these three methods in the primary reformer process are tabulated in Table 5, from which one can readily find that the Multi-DPLS requires more time to train the model because the dimensionality of the augmented input vector is very high. Although the SMR-based soft sensor is also a time-consuming method due to the dichotomy method, the prediction accuracy is much higher than Multi-DPLS.

As for the computational burden based on SMR, we can note that: (1) in the numerical example the input variables are two-dimensional, where the CPT${}_{trn}$ is much less than the primary reformer process of which the input variables are 13-dimensional. This is because as more variables are considered, the larger the size of the precision matrices (whose inversions are involved); (2) the computational burden depends on the number of mixing components, and the more mixing components, the more parameters needing to be learnt, which results in more time for model training; and (3) if the input variables are correlated, the non-diagonal elements of covariance are not equal to zero, leading to more time consumed in inverting the covariance matrix.

## 5. Conclusions

In this paper, with the aim of dealing with outliers when developing soft sensors for multimode industrial processes, we have proposed a robust modeling approach referred to as the Student’s-*t* mixture model (SMR). Our novel contribution is twofold. First, a regressive model structure with finite mixture of Student’s-*t* distributions has been designed, and the corresponding parameter-learning algorithm based on the EM algorithm has also been developed. Second, case studies have been conducted on both numerical and real-word industrial datasets to evaluate the performance of SMR. The results have demonstrated that SMR can handle multimode characteristics well and is more robust against outliers compared to some state-of-the-art methods.

In our future work, two challenging issues are taken into consideration: (1) how to complete the model-selection and parameter-learning tasks without traversing all candidate numbers of mixing components, and without the validating dataset; and (2) how to deal with the performance degradation of the soft sensor caused by time-variation factors. Our solution is to formulate an adaptive Bayesian SMR (BSMR), which randomizes model parameters (including the number of mixing components *K*) and updates the BSMR in a recursive fashion online. 

## Figures and Tables

**Table 1:** Configuration of three Student component.

	k=1	k=2	k=3
$\pi_{k}$	0.2	0.3	0.5
$\mu_{k}$	$\left[\begin{array}{cc} -8 & 1 \end{array}\right]$	$\left[\begin{array}{cc} 4 & 8 \end{array}\right]$	$\left[\begin{array}{cc} 3 & -5 \end{array}\right]$
$\Lambda_{k}$	$\left[\begin{array}{cc} 2.0 & 1.0\\ 1.0 & 1.0 \end{array}\right]$	$\left[\begin{array}{cc} 1.0 & 0.5\\ 0.5 & 2.0 \end{array}\right]$	$\left[\begin{array}{cc} 3.0 & -1.0\\ -1.0 & 1.5 \end{array}\right]$
$\nu_{k}$	3	3	3
$\varphi_{k}$	${\left[\begin{array}{ccc} 1 & 1 & 0 \end{array}\right]}^{T}$	${\left[\begin{array}{ccc} 1 & -1 & 0 \end{array}\right]}^{T}$	${\left[\begin{array}{ccc} -1 & 1 & 2 \end{array}\right]}^{T}$
$\lambda_{k}$	0.25	0.25	0.25

**Table 2:** RMSE of various methods on the validating and testing datasets.

Outliers	Dataset	Multi-DPLS	GMR	SMR
1%	validating	3.9414	1.9097	1.5939
	testing	4.1216	1.6776	1.5208
3%	validating	4.0450	2.0692	1.6398
	testing	4.2969	2.3787	1.5986
5%	validating	4.1307	2.2223	1.7352
	testing	4.3127	2.7476	1.6388

**Table 3:** Average CPT (in second) consumed by various methods for the numerical example.

Outliers	CPT${}_{\mathrm{trn}}$				CPT${}_{\mathrm{tst}}$		
	Multi-DPLS	GMR	SMR		Multi-DPLS	GMR	SMR
1%	0.0283	0.0095	0.0951		0.0013	0.001	0.00072
3%	0.0148	0.0135	0.1087		0.0012	0.0012	0.000846
5%	0.0193	0.0164	0.1099		0.0011	0.001	0.000777

**Table 4:** Descriptions of process variables in the primary reformer.

Tags	Descriptions
FR03001.PV	Flow rate of fuel NG into 03B001
FR03002.PV	Flow rate of fuel off gas into 03B001
PC03002.PV	Pressure of fuel off gas at 03E005’s exit
PC03007.PV	Pressure of furnace flue gas at 03B001’s exit
TI03001.PV	Temperature of fuel off gas at 03E005’s exit
TI03009.PV	Temperature of fuel NG at 03B002E06’s exit
TR03012.PV	Temperature of process gas at 03B001’s entrance
TI03013.PV	Temperature of furnace flue gas at 03B001’s top left
TI03014.PV	Temperature of furnace flue gas at 03B001’s top right
TR03015.PV	Temperature of mixed furnace flue gas at 03B001’s top
TR03016.PV	Temperature of transformed gas at 03B001’s left exit
TR03017.PV	Temperature of transformed gas at 03B001’s right exit
TR03020.PV	Temperature of transformed gas at 03B001’s exit

**Table 5:** Average CPT (in second) consumed by various methods for the primary reformer process.

Time/Method	Multi-DPLS	GMR	SMR
CPT${}_{trn}$	4.4908	0.847	3.002
CPT${}_{tst}$	0.0731	0.009	0.005
